# Our evolved unique pleasure circuit makes humans different from apes: Reconsideration of data derived from animal studies

**DOI:** 10.15761/JSIN.1000191

**Published:** 2018-02-28

**Authors:** Kenneth Blum, Marjorie Gondré-Lewis, Bruce Steinberg1, Igor Elman, David Baron, Edward J Modestino, Rajendra D Badgaiyan, Mark S Gold

**Affiliations:** 1 Department of Psychiatry, Boonshoft School of Medicine, Dayton VA Medical Center, Wright State University, Dayton, OH, USA; 2 Department of Psychiatry, McKnight Brain Institute, University of Florida College of Medicine, Gainesville, FL, USA; 3 Department of Psychiatry and Behavioral Sciences, Keck Medicine University of Southern California, Los Angeles, CA, USA; 4 Division of Applied Clinical Research & Education, Dominion Diagnostics, LLC, North Kingstown, RI, USA; 5 Department of Precision Medicine, Geneus Health LLC, San Antonio, TX, USA; 6 Department of Addiction Research & Therapy, Nupathways Inc., Innsbrook, MO, USA; 7 Department of Clinical Neurology, Path Foundation, New York, NY, USA; 8 Division of Neuroscience-Based Addiction Therapy, The Shores Treatment & Recovery Center, Port Saint Lucie, FL, USA; 9 Institute of Psychology, Eötvös Loránd University, Budapest, Hungary; 10 Division of Addiction Research, Dominion Diagnostics, LLC. North Kingston, RI, USA; 11 Victory Nutrition International, Lederach, PA., USA; 12 National Human Genome Center at Howard University, Washington, DC., USA; 13 Departments of Anatomy and Psychiatry, Howard University College of Medicine, Washington, DC USA; 14 Department of Psychology, Curry College, Milton, MA, USA; 15 Department Psychiatry, Cooper University School of Medicine, Camden, NJ, USA; 16 Department of Psychiatry, Washington University, St. Louis, MO, USA

**Keywords:** brain reward circuitry, comparative neuroanatomy, dopamine, hominids, pleasure, reward deficiency

## Abstract

The brain regions tied to pleasure can be triggered by engaging in sex, eating tasty food, watching a movie, accomplishments at school and athletics, consuming drugs, and noble efforts to help the community, the country, and the world. It is noteworthy that research suggests that the latter type of satisfaction, supporting the community, may result in the most substantial positive effects on our immune system. However, these pathways for these effects are not understood. Berridge and Kringelbach have suggested that pleasure is mediated by well-developed mesocorticolimbic circuitry and serves adaptive functions. In affective disorders, anhedonia (lack of pleasure) or dysphoria (negative affect) can result from a breakdown of that hedonic system. Most importantly, human neuroimaging investigations indicate that surprisingly similar circuitry is activated by quite diverse pleasures, suggesting a common neural pathway shared by all rewarding stimuli and behaviors.

Over many years the controversy of dopamine involvement in pleasure/reward has led to confusion in terms, such as trying to separate motivation from pure pleasure (i.e., wanting versus liking). We take the position that animal studies cannot provide real clinical information that is described by self-reports in humans. On November 23^rd^, 2017, evidence for our concerns was revealed. A brain system involved in everything from addiction to autism appears to have evolved differently in humans than in apes, as reported by a large research team in the journal *Science*. To reiterate, the new findings by Sousa et al., also suggest the importance of not over-relying on rodent and even non-human primate studies. Extrapolations, when it comes to the concept of pleasure, dopamine, and reinforcement, are not supported by these data. Human experience and study are now much more critical and important. Extrapolations from non-humans to humans may be more fiction than fact. While this statement is bold it should not at all suggest that animal date is unimportant, that is not the case. It is extremely valuable in many aspects and we must encourage the development of animal models for disease. However, we must be cautious in our interpretation of results without leaping to conclusions that may be explained by follow-up human experiments and subsequent data.

We are further proposing that in terms of overcoming a never –ending battle related to the current drug epidemic, the scientific community should realize that disturbing dopamine homeostasis by taking drugs or having a system compromised by genes or other epigenetic experiences, should be treated by alternative therapeutic modalities, expressed in this article as a realistic key goal. Application of genetic addiction risk (GARS™) testing and pro-dopamine regulation (KB220) should be considered along with other promising technologies including cognitive behavioral therapy, mind fullness, brain spotting and trauma therapy. Basic scientists have worked very hard to dis-entangle pleasure from incentive salience and learning signals in brain reward circuitry, but this work may be limited to animal models and rodents. A different consideration regarding the human reward systems is required.

## Introduction

We are compelled as neuroscientists and clinicians to provide information regarding the state of brain reward circuitry. Our new understanding must reconsider some data derived from animal studies that tries to dis-entangle pleasure from incentive salience and learning signals in brain reward circuitry.

The brain regions circuitry tied to pleasure are difficult to accurately describe, partly, because of the many different ways we can trigger enjoyment or pleasurable feelings. Pleasure can result from engaging in sex, eating tasty food, watching a movie, accomplishments at school and athletics, consuming drugs, and noble efforts to help the community, the country, and the world. It is noteworthy that research suggests that the latter type of satisfaction, supporting the community, may result in the most substantial positive effects on our immune system, but these pathways for these effects are not understood. In fact, Berridge & Kringelbach [1], suggest that pleasure is mediated by well-developed mesocorticolimbic circuitry and serves adaptive functions. In affective disorders, anhedonia (lack of pleasure) or dysphoria (negative affect) can result from breakdowns of that hedonic system. Most importantly, Blum et al. [2] pointed out in some published works that human neuroimaging investigations indicate that surprisingly similar circuitry is activated by quite diverse pleasures, suggesting a common neural currency shared by all rewarding stimuli and behaviors.

While there is some controversy involving the concepts of “wanting” for reward as proposed [1] and modified by Blum et al. [3] it is generally agreed at least from animal work that “wanting” is generated by a large and distributed brain system. “Liking,” or pleasure itself, is generated by a smaller set of hedonic hot spots within limbic circuitry [4]. It has been stated by Berridge & Kringelbach [1], “*Those hot spots also can be embedded in broader anatomical patterns of valence organization, such as in a keyboard pattern of nucleus accumbens generators for desire versus dread.”*

In contrast, some of the best-known candidates for pleasure/reward generators, including electrodes in the mesolimbic dopamine system, may not generate pleasure alter all. These emerging insights into brain pleasure mechanisms may eventually facilitate better treatments for affective disorders. However, this may not be entirely accurate based on new information related to anatomical differences between non-human primates like Apes and *Homo sapiens*.

In this regard, considering the opposite of pleasure (hedonic), many studies have provided a solid neurochemical and even neurogenetic foundation for a condition termed anhedonia. According to Weis [5] the anhedonia hypothesis – that brain dopamine plays a critical role in the subjective pleasure associated with positive rewards–growing evidence reveals that dopamine plays a critical role in the objective reinforcement and incentive motivation associated with food and water, brain stimulation reward, and psychomotor stimulant and opiate reward. The hypothesis called to attention the apparent paradox that neuroleptics, drugs used to treat a condition involving anhedonia (schizophrenia), attenuated in laboratory animals the positive reinforcement that we usually associate with pleasure. While this holds true for the acute effects of specific drugs of abuse and even powerful non-drug addictive behaviors like gambling, as denoted in Reward Deficiency Syndrome (RDS) [6], the field is remiss for not embracing the bi-directional effects of dopaminergic agents. Acute administration induces increased dopaminergic activity, while chronic use reduces dopamine release at the reward sites of the brain [7].

Despite its limited heuristic value for the understanding of schizophrenia, the anhedonia hypothesis has had a significant impact on biological theories of reinforcement, motivation, and addiction [8]. Brain dopamine plays a considerable role in reinforcement of response habits, caloric intake, conditioned preferences, and the synaptic plasticity in cellular models of learning and memory [9]. Thus, the notion that dopamine plays a dominant role in reinforcement is fundamental to the psychomotor stimulant theory of addiction, to most neuroadaptation theories of addiction, and to current theories of conditioned reinforcement and reward prediction. Properly understood, it is also fundamental to recent theories of incentive motivation [1].

While the concept of “wanting and liking” seems reasonable and plays a significant role in understanding goal-directed motivation and even making someone addictive, its interaction with consciousness must be explored. Anselme & Robinson [10] suggested that most human and animal behaviors emerge from pleasure-seeking and goal-directedness, suggesting that they are primarily under conscious control. However, “wanting” and “liking” are believed to be adaptive core subcortical processes working at an unconscious level and are responsible for guiding behavior toward appropriate rewards. Anselme & Robinson [10] examined whether “wanting” is an inherent property of conscious goals and “liking” an intrinsic component of conscious feelings. They argue that “wanting” and “liking” depend on mechanisms acting below the level of consciousness, explaining why individuals often struggle to enhance or restrain their motivations and emotions using conscious control. In particular, hyperactivity of subcortical “wanting” systems has been tied to pathological behaviors such as drug addiction and gambling disorder. Moreover, in addicts, cognitive processes intended to curb drug-seeking wage a constant battle against subcortical urges to take more drug that often ends in relapse following repeated assaults. In fact, it is believed that in non-pathological contexts, “wanting” and “liking” interact with major cognitive processes to guide goal-directed actions. The complex interaction of sub-cortical processes with conscious, cortical activity makes extrapolation of animal research to human research on pleasure circuits and behavior, difficult.

## Discussion

The following material is dissected into a number of important aspects related to the reward system and pleasure concepts. We take the position that animal studies cannot provide real clinical information that is described by self-reports in humans. On November 23^rd^, 2017, evidence for our concerns was revealed. A brain system involved in everything from addiction to autism appears to have evolved differently in humans than in apes, as reported by a large research team in the journal *Science*. To reiterate, the new findings by Sousa et al., also suggests the importance of not over-relying on rodent and even non-human primate studies. Extrapolations when it comes to the concept of pleasure, dopamine, and reinforcement are not supported by these data. Human experience and study are now much more critical and important. Extrapolations from non-humans to humans may be more fiction than fact. We are further proposing that in terms of overcoming a never –ending battle related to the current drug epidemic, the scientific community should realize that disturbing dopamine homeostasis by taking drugs or having a system compromised by genes or other epigenetic experiences should be treated by alternative therapeutic modalities as expressed in this article as a realistic key goal. Application of genetic addiction risk (GARS) testing and prodopamine regulation (KB220) should be considered along with other promising technologies including cognitive behavioral therapy, mind fullness, brain spotting and trauma therapy. Basic scientists have worked very hard to dis-entangle pleasure from incentive salience and learning signals in brain reward circuitry but this work may be limited to animal models and rodents. A different consideration regarding the human reward systems is required.

## Homeostasis

The first and primary reward function derives from the need of the body to have specific substances for building its structure and maintaining its function. The concentration of these substances and their derivatives is finely regulated and results in homeostatic balance. Most importantly, deviation from specific set points of this balance requires replenishment from substances in our environment including water and food. The existence of hunger and thirst sensations demonstrates that individuals associate the absence of necessary substances with foods and liquids. For example, when the blood sodium concentration exceeds its set point, we drink water, but depletion of sodium leads to ingestion of salt [11]. Concerning reward deficiency and a compromised brain reward circuit, it is agreed that humans will opt to self –medicate or engage in repetitive addictive behaviors that their genetically induced hypodopaminergia requires achieving asymptotic homeostasis.

There are two brain systems that serve to maintain homeostasis. The hypothalamic feeding and drinking centers together with intestinal hormones deal with immediate homeostatic imbalances by rapidly regulating food and liquid intake [12]. In contrast, the reward centers mediate reinforcement for learning and provide advance information for economic decisions and thus can elicit behaviors for obtaining the necessary substances well before homeostatic imbalances and challenges arise. This preemptive function has survival value, since palatable food and liquid may not always be available when an imbalance occurs.

Regarding usual physiological response, homeostatic imbalances are the likely source of hunger and thirst drives whose reduction is considered a prime factor for eating and drinking in drive reduction theories [11,13]. They engage the hypothalamus for immediate alleviation of the imbalances and the reward systems for preventing them. The distinction in psychology between drive reduction for maintaining homeostasis and reward incentives for learning and pursuit may grossly correspond to the separation of neuronal control centers for homeostasis and reward. The neuroscientific knowledge about distinct hypothalamic and reward systems provides essential information for psychological theories about homeostasis and reward.

The need for maintaining homeostatic balance explains the functions of primary rewards. This constitutes the evolutionary origin of brain systems that value stimuli, objects, events, situations, and activities as rewards and mediate the learning, approach, and pleasure. Along these lines, the heuristic value of effects of food, water, psychoactive drugs as well as addictive behaviors depends on dopaminergic activity and possibly net release at the reward site. The function of all non-primary rewards is built into the original function related to homeostasis, even when it comes to the highest rewards.

## Pleasure is a prime reward function

Pleasure is not only one of the three primary reward functions but it also defines reward. As homeostasis explains the functions of only a limited number of rewards, the principal reason why particular stimuli, objects, events, situations, and activities are rewarding may be due to pleasure. This applies first of all to sex and to the primary homeostatic rewards of food and liquid and extends to money, taste, beauty, social encounters and nonmaterial, internally set, and intrinsic rewards. Pleasure, as the primary effect of rewards, drives the prime reward functions of learning, approach behavior, and decision making and provides the basis for hedonic theories of reward function. We are attracted by most rewards and exert intense efforts to obtain them, just because they are enjoyable [10].

Pleasure is a passive reaction that derives from the experience or prediction of reward and may lead to a long-lasting state of happiness. The word happiness is difficult to define. In fact, just obtaining physical pleasure may not be enough. One key to happiness involves a network of good friends. However, it is not obvious how the higher forms of satisfaction and pleasure are related to an ice cream cone, or to your team winning a sporting event. Recent multidisciplinary research, using both humans and detailed invasive brain analysis of animals has discovered some critical ways that the brain processes pleasure [14].

Pleasure as a hallmark of reward is sufficient for defining a reward, but it may not be necessary. A reward may generate positive learning and approach behavior simply because it contains substances that are essential for body function. When we are hungry, we may eat bad and unpleasant meals. A monkey who receives hundreds of small drops of water every morning in the laboratory is unlikely to feel a rush of pleasure every time it gets the 0.1 ml. Nevertheless, with these precautions in mind, we may define any stimulus, object, event, activity, or situation that has the potential to produce pleasure as a reward. In the context of reward deficiency or for disorders of addiction, homeostasis pursues pharmacological treatments: drugs to treat drug addiction, obesity, and other compulsive behaviors. The theory of allostasis suggests broader approaches - such as re-expanding the range of possible pleasures and providing opportunities to expend effort in their pursuit. [15]. It is noteworthy, the first animal studies eliciting approach behavior by electrical brain stimulation interpreted their findings as a discovery of the brain’s pleasure centers [16] which were later partly associated with midbrain dopamine neurons [17–19] despite the notorious difficulties of identifying emotions in animals.

Evolutionary theories of pleasure: The love connection BO:D

Charles Darwin and other biological scientists that have examined the biological evolution and its basic principles found various mechanisms that steer behavior and biological development. Besides their theory on natural selection, it was particularly the sexual selection process that gained significance in the latter context over the last century, especially when it comes to the question of what makes us “what we are,” i.e., human. However, the capacity to sexually select and evolve is not at all a human accomplishment alone or a sign of our uniqueness; yet, we humans, as it seems, are ingenious in fooling ourselves and others–when we are in love or desperately search for it.

It is well established that modern biological theory conjectures that organisms are the result of evolutionary competition. In fact, Richard Dawkins stresses gene survival and propagation as the basic mechanism of life [20]. Only genes that lead to the fittest phenotype will make it. It is noteworthy that the phenotype is selected based on behavior that maximizes gene propagation. To do so, the phenotype must survive and generate offspring, and be better at it than its competitors. Thus, the ultimate, distal function of rewards is to increase evolutionary fitness by ensuring the survival of the organism and reproduction. It is agreed that learning, approach, economic decisions, and positive emotions are the proximal functions through which phenotypes obtain other necessary nutrients for survival, mating, and care for offspring.

Behavioral reward functions have evolved to help individuals to survive and propagate their genes. Apparently, people need to live well and long enough to reproduce. Most would agree that homo-*sapiens* do so by ingesting the substances that make their bodies function properly. For this reason, foods and drinks are rewards. Additional rewards, including those used for economic exchanges, ensure sufficient palatable food and drink supply. Mating and gene propagation is supported by powerful sexual attraction. Additional properties, like body form, augment the chance to mate and nourish and defend offspring and are therefore also rewards. Care for offspring until they can reproduce themselves helps gene propagation and is rewarding; otherwise, many believe mating is useless. According to David E Comings, as any small edge will ultimately result in evolutionary advantage [21], additional reward mechanisms like novelty seeking and exploration widen the spectrum of available rewards and thus enhance the chance for survival, reproduction, and ultimate gene propagation. These functions may help us to obtain the benefits of distant rewards that are determined by our own interests and not immediately available in the environment. Thus the distal reward function in gene propagation and evolutionary fitness defines the proximal reward functions that we see in everyday behavior. That is why foods, drinks, mates, and offspring are rewarding.

There have been theories linking pleasure as a required component of health benefits **salutogenesis**, (salugenesis). In essence, under these terms, pleasure is described as a state or feeling of happiness and satisfaction resulting from an experience that one enjoys. Regarding pleasure, it is a double-edged sword, on the one hand, it promotes positive feelings (like mindfulness) and even better cognition, possibly through the release of dopamine [22]. But on the other hand, pleasure simultaneously encourages addiction and other negative behaviors, i.e., motivational toxicity. It is a complex neurobiological phenomenon, relying on reward circuitry or limbic activity. It is important to realize that through the “Brain Reward Cascade” (BRC) endorphin and endogenous morphinergic mechanisms may play a role [23]. While natural rewards are essential for survival and appetitive motivation leading to beneficial biological behaviors like eating, sex, and reproduction, crucial social interactions seem to further facilitate the positive effects exerted by pleasurable experiences. Indeed, experimentation with addictive drugs is capable of directly acting on reward pathways and causing deterioration of these systems promoting hypodopaminergia [24]. Most would agree that pleasurable activities can stimulate personal growth and may help to induce healthy behavioral changes, including stress management [25]. The work of Esch and Stefano [26] concerning the link between compassion and love implicate the brain reward system, and pleasure induction suggests that social contact in general, i.e., love, attachment, and compassion, can be highly effective in stress reduction, survival, and overall health.

Understanding the role of neurotransmission and pleasurable states both positive and negative have been adequately studied over many decades [26–37], but comparative anatomical and neurobiological function between animals and homo *sapiens* appear to be required and seem to be in an infancy stage.

## Finding happiness is different between apes and humans

As stated earlier in this expert opinion one key to happiness involves a network of good friends [38]. However, it is not entirely clear exactly how the higher forms of satisfaction and pleasure are related to a sugar rush, winning a sports event or even sky diving, all of which augment dopamine release at the reward brain site. Recent multidisciplinary research, using both humans and detailed invasive brain analysis of animals has discovered some critical ways that the brain processes pleasure.

Remarkably, there are pathways for ordinary liking and pleasure, which are limited in scope as described above in this commentary. However, there are many brain regions, often termed hot and cold spots, that significantly modulate (increase or decrease) our pleasure or even produce the opposite of pleasure— that is disgust and fear [39]. One specific region of the nucleus accumbens is organized like a computer keyboard, with particular stimulus triggers in rows— producing an increase and decrease of pleasure and disgust. Moreover, the cortex has unique roles in the cognitive evaluation of our feelings of pleasure [40]. Importantly, the interplay of these multiple triggers and the higher brain centers in the prefrontal cortex are very intricate and are just being uncovered.

## Desire and reward centers

**Figure 1.**
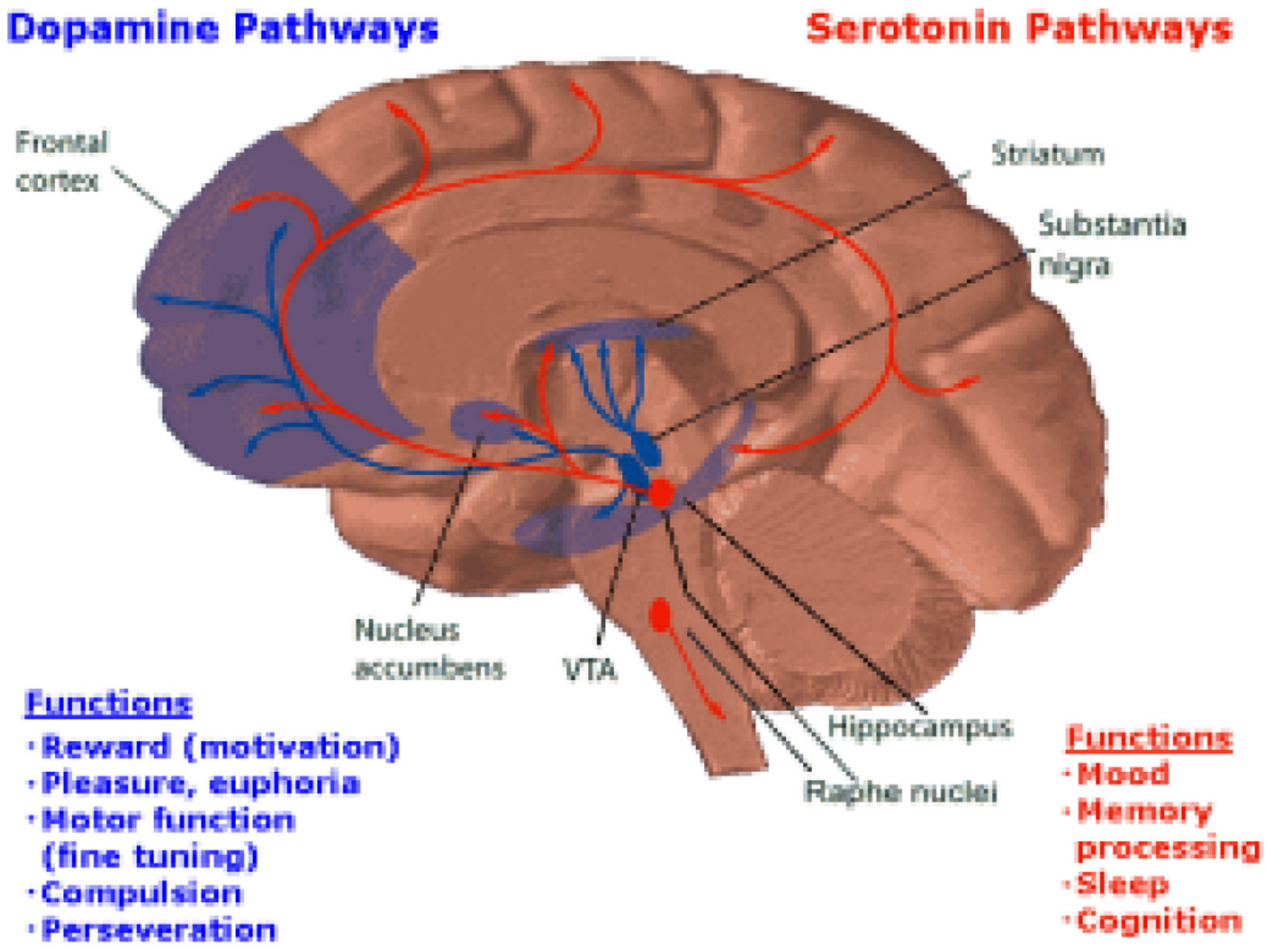
Dopamine and serotonin pathways within the Brain Reward Cascade (BRC) and their functions. Acessed from internet.

It is surprising that many different sources of pleasure activate the same circuits between the mesocorticolimbic regions (Figure 1). Reward and desire are two aspects pleasure induction and have a very widespread, large circuit. Some part of this circuit distinguishes between desire and dread. The so-called pleasure circuitry called “REWARD” involves a well-known dopamine pathway in the mesolimbic system that can influence both pleasure and motivation.

**Figure 2.**
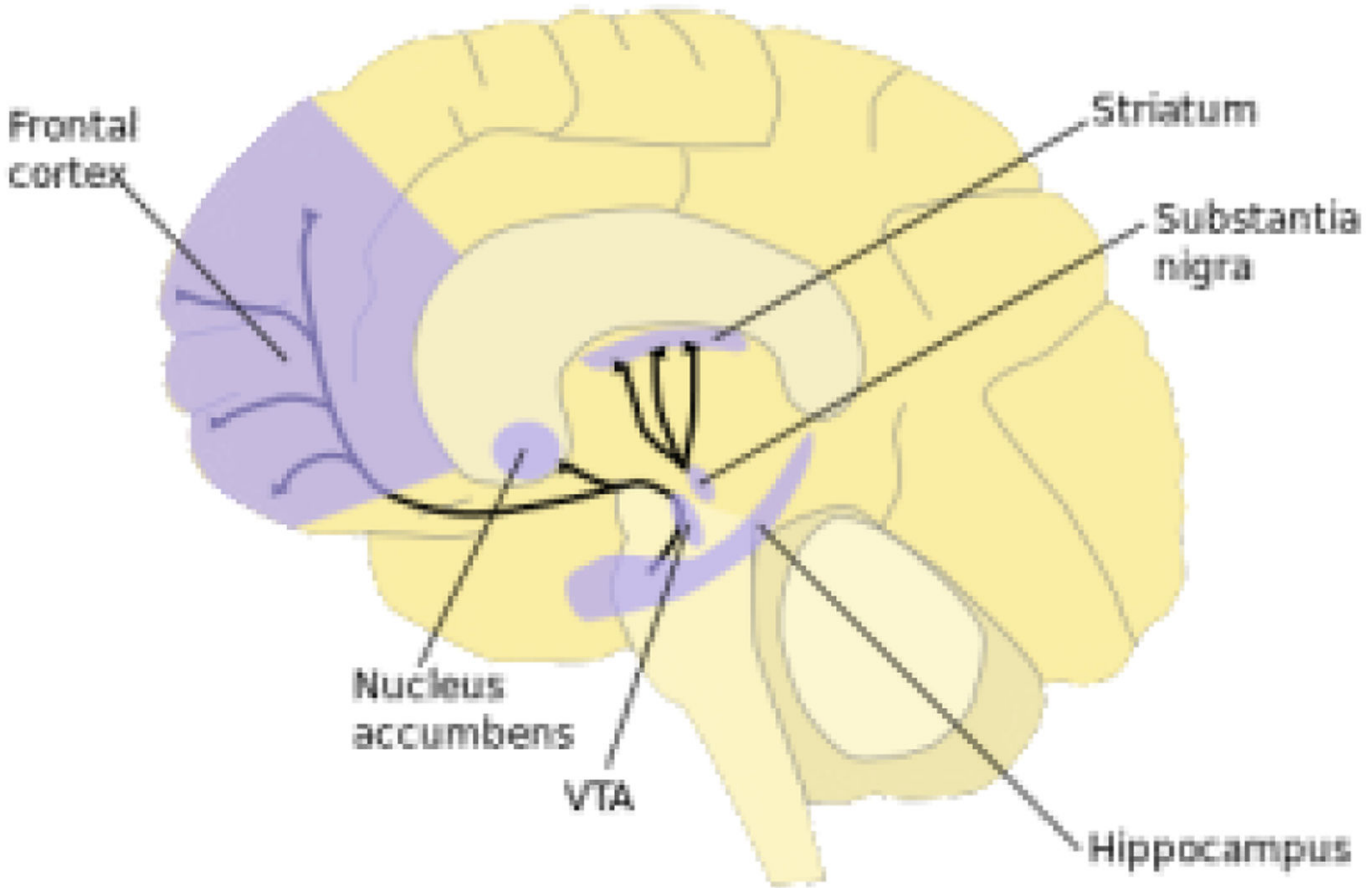
Key brain dopamine-related regions within the Brain Reward Cascade (BRC). Acessed from internet.

In simplest terms, the well-established mesolimbic system is a dopamine circuit for reward. It starts in the ventral tegmental area (VTA) of the midbrain and travels to the nucleus accumbens (Figure 2). It is the cornerstone target to all addictions. The VTA is encompassed with neurons using glutamate, GABA, and dopamine. The nucleus accumbens (NAc) is located within the ventral striatum and is divided into two sub-regions—the motor and limbic regions associated with its core and shell, respectively. The NAc has spiny neurons that receive dopamine from the VTA and glutamate (a dopamine driver) from the hippocampus, amygdala and medial prefrontal cortex. Subsequently, the NAc projects GABA signals to an area termed the ventral pallidum (VP). The region is a relay station in the limbic loop of the basal ganglia, critical for motivation, behavior, emotions and the “Feel Good” response. This defined system of the brain is involved in all addictions –substance, and non –substance related. In 1995, our laboratory coined the term “Reward Deficiency Syndrome” (RDS) to describe genetic and epigenetic induced hypodopaminergia in the “Brain Reward Cascade” that contribute to addiction and compulsive behaviors [3,6,41].

Furthermore, ordinary “liking” of something, or pure pleasure, is represented by small regions mainly in the limbic system (old reptilian part of the brain). These may be part of larger neural circuits. In Latin, *hedus* is the term for “sweet”; and in Greek, *hodone* is the term for “pleasure.” Thus, the word *Hedonic* is now referring to various subcomponents of pleasure: some associated with purely sensory and others with more complex emotions involving morals, aesthetics, and social interactions. The capacity to have pleasure is part of being healthy and may even extend life, especially if linked to *optimism* as a dopaminergic response [42].

Psychiatric illness often includes symptoms of an abnormal inability to experience pleasure, referred *to as anhedonia*. A negative feeling state is called *dysphoria*, which can consist of many emotions such as pain, depression, anxiety, fear, and disgust. Previously many scientists used animal research to uncover the complex mechanisms of pleasure, liking, motivation and even emotions like panic and fear, as discussed above [43]. However, as a significant amount of related research about the specific brain regions of pleasure/reward circuitry has been derived from invasive studies of animals, these cannot be directly compared with subjective states experienced by humans.

In an attempt to resolve the controversy regarding the causal contributions of mesolimbic dopamine systems to reward, we have previously evaluated the three-main competing explanatory categories: “liking,” “learning,” and “wanting” [3]. That is, dopamine may mediate (a) liking: the hedonic impact of reward, (b) learning: learned predictions about rewarding effects, or (c) wanting: the pursuit of rewards by attributing incentive salience to reward-related stimuli [44]. We have evaluated these hypotheses, especially as they relate to the RDS, and we find that the incentive salience or “wanting” hypothesis of dopaminergic functioning is supported by a majority of the scientific evidence. Various neuroimaging studies have shown that anticipated behaviors such as sex and gaming, delicious foods and drugs of abuse all affect brain regions associated with reward networks, and may not be unidirectional. Drugs of abuse enhance dopamine signaling which sensitizes mesolimbic brain mechanisms that apparently evolved explicitly to attribute incentive salience to various rewards [45].

Addictive substances are voluntarily self-administered, and they enhance (directly or indirectly) dopaminergic synaptic function in the NAc. This activation of the brain reward networks (producing the ecstatic “high” that users seek). Although these circuits were initially thought to encode a set point of hedonic tone, it is now being considered to be far more complicated in function, also encoding attention, reward expectancy, disconfirmation of reward expectancy, and incentive motivation [46]. The argument about addiction as a disease may be confused with a predisposition to substance and nonsubstance rewards relative to the extreme effect of drugs of abuse on brain neurochemistry. The former sets up an individual to be at high risk through both genetic polymorphisms in reward genes as well as harmful epigenetic insult. Some Psychologists, even with all the data, still infer that addiction is not a disease [47]. Elevated stress levels, together with polymorphisms (genetic variations) of various dopaminergic genes and the genes related to other neurotransmitters (and their genetic variants), and may have an additive effect on vulnerability to various addictions [48]. In this regard, Vanyukov, *et al.* [48] suggested based on review that whereas the gateway hypothesis does not specify mechanistic connections between “stages,” and does not extend to the risks for *addictions* the concept of common liability to addictions may be more parsimonious. The latter theory is grounded in genetic theory and supported by data identifying common sources of variation in the risk for specific addictions (e.g., RDS). This commonality has identifiable neurobiological substrate and plausible evolutionary explanations.

Over many years the controversy of dopamine involvement in especially “pleasure” has led to confusion concerning separating motivation from actual pleasure (wanting versus liking) [49]. We take the position that animal studies cannot provide real clinical information as described by self-reports in humans. As mentioned earlier and in the abstract, on November 23rd, 2017, evidence for our concerns was discovered [50]

In essence, although nonhuman primate brains are similar to our own, the disparity between other primates and those of human cognitive abilities tells us that surface similarity is not the whole story. Sousa ***et al.*** [50] small case found various differentially expressed genes, to associate with pleasure related systems. Furthermore, the dopaminergic interneurons located in the human neocortex were absent from the neocortex of nonhuman African apes. Such differences in neuronal transcriptional programs may underlie a variety of neurodevelopmental disorders.

In simpler terms, the system controls the production of dopamine, a chemical messenger that plays a significant role in pleasure and rewards. The senior author, Dr. Nenad Sestan from Yale, stated: *“Humans have evolved a dopamine system that is different than the one in chimpanzees.”* This may explain why the behavior of humans is so unique from that of non-human primates, even though our brains are so surprisingly similar, Sestan said: *“It might also shed light on why people are vulnerable to mental disorders such as autism (possibly even addiction).”* Remarkably, this research finding emerged from an extensive, multicenter collaboration to compare the brains across several species. These researchers examined 247 specimens of neural tissue from six humans, five chimpanzees, and five macaque monkeys. Moreover, these investigators analyzed which genes were turned on or off in 16 regions of the brain. While the differences among species were subtle, there was a remarkable contrast in the neocortices, specifically in an area of the brain that is much more developed in humans than in chimpanzees. In fact, these researchers found that a gene called *tyrosine hydroxylase* (TH) for the enzyme, responsible for the production of dopamine, was expressed in the neocortex of humans, but not chimpanzees. As discussed earlier, dopamine is best known for its essential role within the brain’s reward system; the very system that responds to everything from sex, to gambling, to food, and to addictive drugs. However, dopamine also assists in regulating emotional responses, memory, and movement. Notably, abnormal dopamine levels have been linked to disorders including Parkinson’s, schizophrenia and spectrum disorders such as autism and addiction or RDS.

Nora Volkow, the director of NIDA, pointed out that one alluring possibility is that the neurotransmitter dopamine plays a substantial role in humans’ ability to pursue various rewards that are perhaps months or even years away in the future. This same idea has been suggested by Dr. Robert Sapolsky, a professor of biology and neurology at Stanford University. Dr. Sapolsky cited evidence that dopamine levels rise dramatically in humans when we anticipate potential rewards that are uncertain and even far off in our futures, such as retirement or even the possible alterlife. This may explain what often motivates people to work for things that have no apparent short-term benefit [51]. In similar work, Volkow and Bale [52] proposed a model in which dopamine can favor NOW processes through phasic signaling in reward circuits or LATER processes through tonic signaling in control circuits. Specifically, they suggest that through its modulation of the orbitofrontal cortex, which processes salience attribution, dopamine also enables shilting from NOW to LATER, while its modulation of the insula, which processes interoceptive information, influences the probability of selecting NOW versus LATER actions based on an individual’s physiological state. This hypothesis further supports the concept that disruptions along these circuits contribute to diverse pathologies, including obesity and addiction or RDS.

## Summary

Sousa ***et al.*** [50] also found differences in much older areas, including an ancient structure called the cerebellum. Accordingly, an ancient part of the human brain seems to have very recent change. It will take years to understand more fully what all the changes mean, but this finding could eventually help divulge what makes the human brain unique, and even what goes wrong in a range of brain disease states. The role of dopamine in brain function has been well established throughout many decades of research and merited the Nobel Prize in 2000. Continued work by one of us (KB) and the late Ernest P. Noble, showed the role of dopamine genetics in severe alcoholism. Also work by Mark Gold and Charles Dackis with regard to the “dopamine depletion hypothesis” and cocaine, as well the work of Elman *et al.* on both RDS and anti-reward, suggest the real need for balancing brain dopamine to induce homeostasis [53–56]. The new findings by Sousa *et al.*, [50] also call for the importance of dopamine homeostasis through genetic addiction risk (GARS) testing and Pro-dopamine regulation (KB220PAM), as pointed out by Gold and associates many years ago [57–59]. While we applaud the elegant work of Berridge and associates in disentangling pleasure from incentive salience and learning signals in brain reward circuitry in animal models, new consideration especially as it relates to humans is required.
